# Determinants of Psychosocial Difficulties Experienced by Persons with Brain Disorders: Towards a ‘Horizontal Epidemiology’ Approach

**DOI:** 10.1371/journal.pone.0141322

**Published:** 2015-12-16

**Authors:** Carla Sabariego, Michaela Coenen, Carolina Ballert, Maria Cabello, Matilde Leonardi, Marta Anczewska, Tuuli Pitkänen, Alberto Raggi, Blanca Mellor, Venusia Covelli, Piotr Świtaj, Jonna Levola, Silvia Schiavolin, Anna Chrostek, Jerome Bickenbach, Somnath Chatterji, Alarcos Cieza

**Affiliations:** 1 Institute for Medical Information Processing, Biometry and Epidemiology (IBE), Chair for Public Health and Health Services Research, Research Unit for Biopsychosocial Health, Ludwig-Maximilians-University (LMU) Munich, Munich, Germany; 2 Swiss Paraplegic Research, Nottwil, Switzerland; 3 Department of Psychiatry, Universidad Autónoma de Madrid, Psychiatry Service, Instituto de Investigación del Hospital Universitario de La Princesa (IIS-IP), Instituto de Salud Carlos III, Centro de Investigación Biomédica en Red de Salud Mental (CIBERSAM), Madrid, Spain; 4 Department of Psychiatry, Universidad Autónoma de Madrid, Madrid, Spain; 5 Neurology, Public Health and Disability Unit, Scientific Directorate, Neurological Institute Carlo Besta (IRCCS) Foundation, Milan, Italy; 6 Department of Psychiatry, Institute of Psychiatry and Neurology, Warsaw, Poland; 7 A-Clinic Foundation, Helsinki, Finland; 8 Department of Mental Health and Substance Abuse Services, National Institute for Health and Welfare, Helsinki, Finland; 9 Division of Neurosurgery II, Neurological Institute Carlos Besta (IRCCS) Foundation, Milan, Italy; 10 Department of Measurement and Health Information Systems, Multi-Country Studies, World Health Organization, Geneva, Switzerland; 11 Faculty of Social and Human Sciences, School of Psychology, University of Southampton, Southampton, United Kingdom; Zhejiang Key Laborotory for Research in Assesment of Cognitive Impairments, CHINA

## Abstract

**Background:**

Persons with brain disorders experience significant psychosocial difficulties (PSD) in daily life, e.g. problems with managing daily routine or emotional lability, and the level of the PSD depends on social, physical and political environments, and psychologic-personal determinants. Our objective is to determine a brief set of environmental and psychologic-personal factors that are shared determinants of PSD among persons with different brain disorders.

**Methods:**

Cross-sectional study, convenience sample of persons with either dementia, stroke, multiple sclerosis, epilepsy, migraine, depression, schizophrenia, substance dependence or Parkinson’s disease. Random forest regression and classical linear regression were used in the analyses.

**Results:**

722 subjects were interviewed in four European countries. The brief set of determinants encompasses presence of comorbidities, health status appraisal, stressful life events, personality changes, adaptation, self-esteem, self-worth, built environment, weather, and health problems in the family.

**Conclusions:**

The identified brief set of common determinants of PSD can be used to support the implementation of cross-cutting interventions, social actions and policy tools to lower PSD experienced by persons with brain disorders. This set complements a recently proposed reliable and valid direct metric of PSD for brain disorders called PARADISE24.

## Introduction

Brain disorders, such as dementia, stroke, Parkinson’s Disease (PD), depression and schizophrenia, are mostly chronic neurological or psychiatric disorders of long duration which exert a high degree of burden on daily life. Affected persons often react with distress when receiving the diagnosis, have difficulties living with the illness, are often forced to dramatically change their lifestyles and are at risk of being stigmatised [1]. Persons with brain disorders also experience significant psychosocial difficulties (PSD), ranging from problems with attention and memory, emotional lability and listlessness to problems with managing their daily routines, problems interacting with significant others and difficulties at work [2–6].

**Fig 1:**
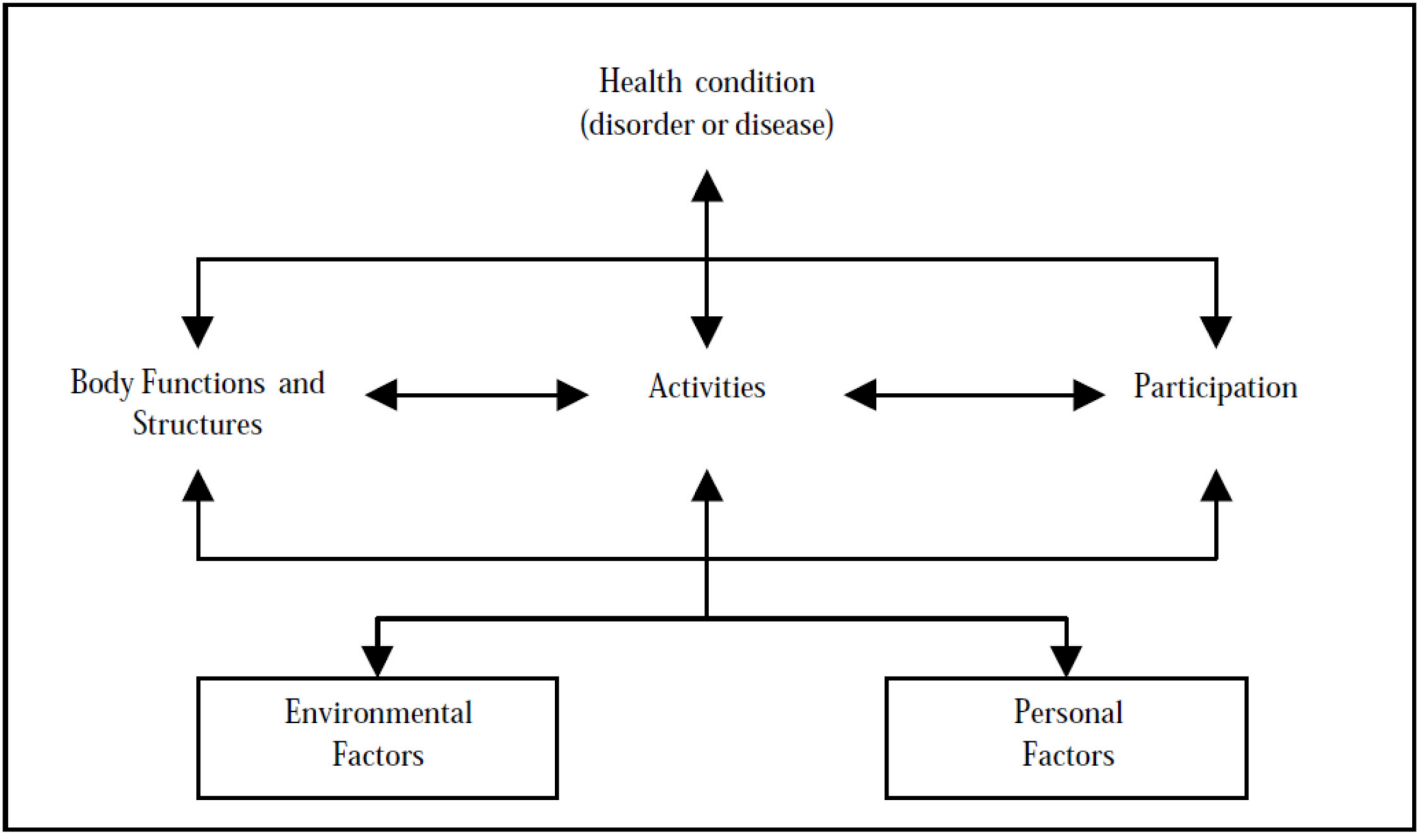
The biopsychosocial model proposed in the International Classification of Functioning, Disability and Health (ICF).

The severity of these PSDs depends on many factors in the close and extended environments, including social, physical and political environments. This understanding coincides with the biopsychosocial model proposed in the International Classification of Functioning, Disability and Health (ICF) (Fig 1), where functioning is defined as a continuum encompassing body functions and structures and activities and participation and determined by interactions between health conditions and contextual factors (environmental and personal factors) [7, 8]. According to the ICF model, PSDs have recently been specifically defined as impairments in mental functions and body functions under nervous-system control, activity limitations and participation restrictions resulting from interactions among the brain disorder, the environment and personal factors [9]. In this sense, living in the countryside, the availability of adequate treatment, having a supportive work environment, high self-esteem or strong self-confidence and successful coping strategies are examples of environmental and personal factors which definitely determine the intensity of PSD accompanying a brain disorder.

In the ICF, environmental factors are defined in a very inclusive way, going far beyond the built environment and encompassing factors such as health-service provision or quality of the social environment. Formally, environmental factors are divided into five groups in the ICF: [1] products and technology, [2] the natural environment, [3] support and relationships, [4] attitudes of others, and [5] services, systems and policies [8]. Environmental factors include medication and built environment, weather and climate, support provided by the family and by health professionals, being treated with respect by family and friends and the provision of health-care services. Personal factors have not yet been classified in the ICF, but were previously tentatively defined as “*psychologic-personal factors*” and subdivided into seven groups: [1] socio-demographic personal characteristics, [2] position in the immediate social and physical context, [3] personal history and biography, [4] feelings, [5] thoughts and beliefs, [6] motives and [7] patterns of experience and behaviour [10]. According to this categorization, psychologic-personal factors include age, gender, being a caregiver, having experienced stressful life events, knowledge and opinions about one’s own disease and interest in sports.

According to the rationale of the ICF model, there is no reason to believe that determinants of PSD are not shared across brain conditions. This hypothesis has recently been challenged, and it has been clearly shown that several determinants of PSD, i.e. medication, assistance from family and their attitudes and care from health professionals and their attitudes are common across brain disorders [9]. A recent review of qualitative studies has also identified eight determinants shared among persons with very different brain disorders, such as substance dependence, depression, epilepsy and multiple sclerosis (MS). In this work, stages in life and associated roles, the presence of trusting or supportive informal and formal relationships, social inclusion, availability of work opportunities and self-help groups, self-determination, contact with motivated professionals and achieving a balance between protection and overprotection were reported as shared determinants [11].

Knowledge of determinants of PSD across brain disorders is essential to provide health professionals and policy makers with intervention targets and patients and their families with insights about what can be done beyond pharmaceutical treatment to improve their lives. Effective treatment strategies are available for some brain disorders, but usually focus on disease control and reduction of mostly acute symptoms. Due to the chronic, sometimes continuous, sometimes degenerating character of brain disorders and the experienced PSDs, broader strategies extending beyond merely targeting symptoms through medical interventions and focusing on environmental aspects and personal factors are required. It is essential to achieve a better understanding of the determinants of PSD to be able to design effective and sound interventions targeting environmental and personal factors and to eventually improve the lives of persons with brain disorders. Since these persons experience common PSDs, a thorough understanding of the determinants of PSD across brain disorders is indispensable for developing effective and efficient cross-cutting strategies.

Determinants of PSD experienced by persons with brain disorders in daily life have been seldom considered in research. The available literature on the determinants of brain disorders focuses mainly on factors related to the aetiology of these disorders, such as genetics, biomedical factors and environmental factors. Although efforts to identify risk factors to prevent the emergence of brain disorders are undeniably essential, we must not forget the reasons why brain disorders are among the most disabling of disorders. They are often chronic, some start very early in life and have a meaningful cumulative impact on several aspects of daily life [12]. Furthermore, available information on the determinants of PSD is rare and traditionally assessed from a disease-specific perspective. Although it is possible to search for shared determinants in disease-specific publications, the comparability of the obtained information is always limited because of the different methodologies or different assessment instruments applied. It is essential to directly identify a set of determinants relevant across brain disorders and suitable for guiding the development of cross-cutting interventions. Since the description and assessment of PSDs is only meaningful if done in the context of their determinants, this set would complement a recently proposed reliable and valid direct metric of PSD for brain disorders called PARADISE24 [13].

The objective of this paper is to determine a brief set of environmental and psychologic-personal factors that are determinants of PSD experienced by persons with brain disorders. The routine inclusion of this set in data collection could widen knowledge about the determinants of burden across brain disorders, going beyond acknowledged determinants such as age or disease severity, and guide resource-allocation efforts towards developing and implementing effective interventions to reduce the burden experienced by persons with brain disorders and provide health professionals, policy makers, affected persons and their families with meaningful intervention targets. To our knowledge, this is the first work specifically targeting determinants of PSD across brain disorders.

## Methods

### Design and sample

This was a multi-centre, cross-sectional study carried out in the scope of the project PARADISE (Psychosocial fActors Relevant to BrAin DISorders in Europe, www.paradiseproject.eu) and involving four European study sites [9]. This project was funded under the FP7 by the European Commission and had as a primary goal testing the hypothesis of commonalities regarding PSD and determinants of PSD across brain disorders, i.e. that there are PSDs—defined as impairments of mental functions and impairments of body functions under central nervous system control, activity limitations and participation restrictions—and determinants of PSD that are common in people with brain disorders despite variations in symptomatology, aetiology, and the biochemical basis of their disorders. In the cross-sectional study a convenience sample of 722 persons with dementia (N = 80), stroke (N = 80), multiple sclerosis (MS) (N = 80), epilepsy (N = 80), migraine (N = 80), Parkinson’s Disease (PD) (N = 80), depression (N = 81), schizophrenia (N = 81) or and substance dependency (N = 80) was included. Participants with stroke, MS, epilepsy, migraine and PD were recruited at the Neurological Institute Carlo Besta IRCCS Foundation in Milan, Italy; participants with dementia and schizophrenia at the Institute of Psychiatry and Neurology in Warsaw, Poland; participants with depression at the teaching hospital La Princesa in Madrid, Spain; and participants with substance-dependency at the Järvenpää Addiction Hospital in Haarajoki, Finland. Participants were interviewed by trained clinical researchers using the PARADISE data collection protocol which included 64 PSDs and 59 PSDs determinants of PSDs considered to be common across brain disorders as well as questions targeting demographic information, age, the impact of comorbidities, and disease severity. Individuals participating in the study had to meet the following general inclusion criteria: age ≥ 18 years; main diagnosis (according to ICD-10) of one of the disorders listed above; and the individual had been informed of the purpose and rationale of the study and had signed the “patient consent form”. Further information about the study and the PARADISE protocol is reported in detail elsewhere [13]. The study was conducted in conformity with the ethical principles of the European Commission Research Ethics Committee. The study and the informed consent used were approved by the Ethics Committee of the Ludwig-Maximilian University of Munich, as well as by the Ethics Committees of the Neurological Institute Carlo Besta IRCCS Foundation in Milan, Italy, the Institute of Psychiatry and Neurology in Warsaw, Poland, the teaching hospital La Princesa of the University of Madrid in Madrid, Spain and the Järvenpää Addiction Hospital in Haarajoki, Finland. Individuals participating in the study were informed of the purpose and rationale of the study by a recruiting nurse and signed a patient consent form approved by the Ethics Committees.

### Data analysis

Descriptive statistics were used to characterize the sample. Environmental and psychologic-personal factors included in the PARADISE protocol were split into three groups: *“psychologic-personal factors I”*, including sociodemographic characteristics, position in the immediate social and physical context and personal history and biographic information, *“psychologic-personal factors II”*, including information on thoughts and beliefs and patterns of experience and behaviour, and *“environmental factors”*. Detailed information on all determinants is provided elsewhere [9].

In the *“psychologic-personal factors I”* group, items addressing factors such as disease duration, health-care use, health appraisal and the living and working situation were included. Besides these variables, four Patient Reported Outcome (PRO) tools were included. The impact of comorbidities was assessed using the Self-Reported Comorbidities Questionnaire (SCQ) [14], from which a summary score is derived by adding up to three points obtained from each of the reported health conditions: one point for its presence, one if treatment is received and one if it causes decrements in functioning. Higher scores indicate a higher comorbidity impact. Quality of life was assessed using eight questions from the WHO Quality of Life (WHOQOL-8) questionnaire [15] and social support was evaluated using the Oslo Social Support Scale (OSS) [16]. Stressful life events were assessed with the Social Readjustment Rating Scale (SRRS), and an index reflecting the number of events experienced in the past year was created [17].

In the *“psychologic-personal factors II”* group, items addressing factors such as personality changes, self-worth, and personal beliefs and adaptation, were included. Three patient-reported outcome (PRO) instruments were also included: the Big Five Inventory [18], the Brief Resilient Coping Scale [18] and two items from the General Self-Efficacy Scale (GSES) [19], which were recently identified as sufficient to measure the concept in a psychometric study applying Rasch analysis [20].

“*Environmental factors*” included items addressing factors such as facilitating and hindering aspects of medication, the built environment, public transportation, weather or climate, attitudes and support given by others. Two questions were stated for all items: “*To what extent does/do … have a positive influence on your difficulties*?” and “*To what extent does/do … have a negative influence on your difficulties*?”. The Jefferson Scale of Patient Perceptions of Physician Empathy [21] was also used to assess physicians’ empathy.

The PARADISE24 metric, our dependent variable, provides a single score to estimate the impact of brain disorders on people’s lives, and was developed using Rasch analyses [9]. This metric ranges from zero (no PSDs) to 100 (extreme PSDs). We estimated Spearman correlation coefficients between each of the 59 determinants and the PARADISE24 score to come up with a pre-selection of the most relevant determinants of PSD across brain disorders. Determinants were selected for further analyses if the association was >0.20. This rather conservative cut-off was selected having in mind that correlations of 0.2 may already be regarded as noteworthy and demonstrate some relationship. In setting this cut-off, we intended to reduce the number of predictors but at the same time keep all determinants which had at least some association with the outcome and might become significant predictors.

The most relevant determinants of PSD were then identified by means of random forest regression using the PARADISE24 score as the dependent variable. This regression method can handle a large number of predictors, even in the presence of multicollinearity, and provide unbiased variable importance estimates. The random forest provides a ranking of importance of the independent variables included in the model with regard to their explanatory value for a response variable [10].

The determinants of PSD included in the analyses were also split into three groups: “*psychologic-personal factors I*”, “*psychologic-personal factors II*” and “*environmental factors*”. A random forest regression was carried out separately for each group. Using the resulting ranking of importance, the increase in explained variance was calculated for each random forest regression with a classical linear regression analysis [22]. Starting with the most important variable in each group-specific regression, variables of the three random forests were included stepwise in a final single model, and the changes in the coefficient of determination (R^2^) were observed. Cook’s D was used to detect eventual outliers. A brief set of the most important determinants was selected based on the change in R^2^ curves and the p-value of the regression analyses. The cforest function from the R-package “party” estimated the random forests [23–25]. This analysis included only individuals with less than 40% missing values, which were imputed with the R-package missForest [26]. Regression analyses were controlled for age, gender and level of education.

Data analyses were performed in SPSS, SAS and R.

## Results

### Sample

Altogether 722 subjects were interviewed. Due to cognitive limitations, interviews were carried out with proxies for eight persons with depression, two with stroke and 21 with dementia. The mean interview duration ranged from 51.05 minutes in migraine to 133.97 minutes in dementia. The characteristics of the included population are reported in Table 1.

**Table 1:** Characteristics of the included population.

		Depression	Epilepsy	Migraine	Multiple Sclerosis	Parkinson	Stroke	Schizophrenia	Dementia	Substance dependence
Sample size	N	81	80	80	80	80	80	81	80	80
Age in years	Mean (SD)	54.81 (14.73)	41.23 (11.99)	44.54 (12.12)	41.03 (8.74)	61.24 (10.45)	59.84 (14.36)	38.38 (14.03)	81.03 (5.49)	39.56 (13.15)
Gender	Female(%)	82.7	50.0	86.3	65.0	40.0	43.8	53.1	78.8	37.5
Disease duration in years	Mean (SD)	12.63 (11.57)	18.67 (12.32)	21.13 (14.60)	7.66 (6.94)	6.26 (4.40)	4.00 (6.48)	13.03 (11.83)	3.69 (2.70)	12.16 (8.67)

Overall, 44 determinants of PSD had an association with the PARADISE24 score higher than >0.20 (data not shown) and were included in further analyses (Table 2).

**Table 2:** Questions or questionnaires used to assess the determinants of psychosocial difficulties (PSD) selected for further analyses and split into three groups: “psychologic-personal factors I”, “psychologic-personal factors II” and “environmental factors.”

Determinant (Questions or questionnaires)
**Control variables**
Age
Level of education
Gender
**Psychologic-personal factors I**
Disease duration (Since when have you had the <health condition>?)
Number of visits to a medical doctor (How many visits did you make to a medical doctor (GP and specialist) during the past 12 months due to your < health condition >?)
***Comorbidities (Self-Reported Comorbidities Questionnaire)***
Quality of life (WHOQOL)
***In general*, *how would you rate your health today*?**
Living situation (What is your general living situation?)
Working situation (What is your current working situation?)
Care-giving role (Over the last 12 months, have any members of your household, adults or children, needed your care or support for any reason? This could include financial, physical, emotional, health or personal care or support)
***Stressful life events (Stressful events Index)***
**Psychologic-personal factors II**
Personality—Neuroticism (Big Five Inventory—Neuroticism)
***Personality—Do you think that you have become a different kind of person because of your < health condition >*?**
Resilience (Brief Resilience Scale)
***Self-worth (To what extent do you see yourself as equally worthwhile and deserving as other people*?*)***
Sense of belonging (To what extent do you feel like you belong when attending activities in the community?)
Personal beliefs (To what extent do your personal beliefs give you the strength to face difficulties?)
Self-efficacy (To what extent are you confident you can find the means and ways to get what you want if someone opposes you?)
Self-efficacy (To what extent are you confident that you could deal efficiently with unexpected events?)
***Self-esteem (To what extent do you think that many people view < health condition > or problems related to your < health condition > as something that lessens your value as a person*?*)***
***Adaptation (Generally*, *have you learned to live with (do you accept) the problems and difficulties your <health condition > has led to in your life*?*)***
***Adaptation (Generally*, *do you think that the people around you have come to terms with your problems and difficulties*?*)***
**Environmental factors**
To what extent do side effects of medications make your difficulties worse?
The medication you receive (+)^§^(–)^#^
***The way the environment around you is*** (+) (-)
The way public transportation works (+) (-)
***Weather or climate*** (+) (-)
The awareness of HC that people around you have in general (+) (-)
The help and assistance you receive from your family (+) (-)
The help and assistance you receive from your friends (+) (-)
The help and assistance of your peers or colleagues (+) (-)
The care from health professionals (+) (-)
***The health problems of other family members*** (+) (-)

^§^ (+): positive influence of determinant on PSD.

^#^ (-): negative influence of determinant on PSD.

**Fig 2:**
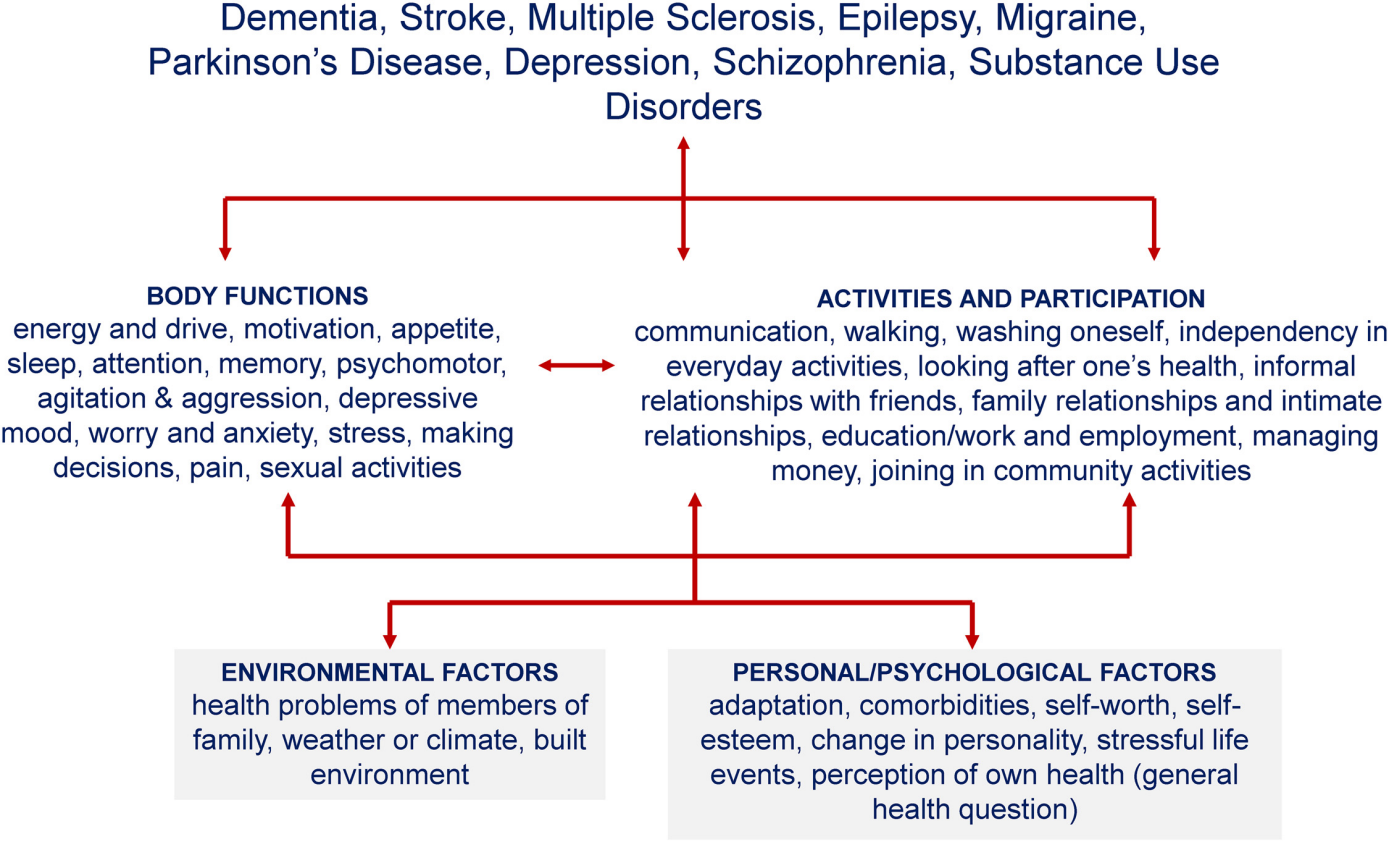
Summary of health conditions and the identified determinants (environmental and psychologic-personal factors). * The psychosocial difficulties included in the PARADISE24 metric and used to identify the determinants are: energy and drive, motivation, appetite, sleep, attention, memory, psychomotor difficulties, agitation & aggression, depressive mood, worry and anxiety, stress, making decisions, pain, sexual activities (body-function domains); communication, walking, washing oneself, independence in everyday activities, looking after one’s health, informal relationships with friends, family and intimate relationships, education/work and employment, managing money, joining in community activities (activities-and-participation domains).

Results of the random forest and linear regressions are reported in Table 3. For each determinant group, the first two columns report the importance estimate and the ranking of importance estimated with random forest regression, respectively. Columns R^2^ adjusted and R^2^ report the increase in explained variance calculated with classical linear regression analysis by adding the determinants stepwise in rank order to the model. Using the obtained forest-based ranking of importance for the three groups and the increase in the explained variance (R^2^ adjusted), 24 determinants were selected for the final random forest and linear models (final regression analysis column). These results identified the 11 most important determinants: three factors from the psychologic-personal factors I group (presence of comorbidities, personal appraisal of health status and experience of stressful life events), five variables from the psychologic-personal factors II group (changes in personality, adaptation on the part of the persons and of others, self-esteem and self-confidence) and three environmental factors (built environment, weather or climate, and health problems of members of the family). Fig 2 summarizes the health conditions, the PSD included in the PARADISE24 metric, and the identified determinants in the present study.

**Table 3:** Results of the random forest and linear regressions used to identify the most important determinants of psychosocial difficulties (PSD). The PARADISE24 score, a single metric score estimating the level of PSD by using Rasch analyses, was used as the dependent variable. Determinants of PSD included in the analyses as independent variables were split into three groups: “psychologic-personal factors I”, “psychologic-personal factors II”, and “environmental factors”. For each group, the first two columns report the importance estimate and the ranking of importance estimated with random forest regression, respectively. The columns R^2^ adjusted and R^2^ report the increase in explained variance calculated with classical linear regression analysis by adding the determinants stepwise in rank order into the model. The dependent variable was the PSD. All models were controlled for age, gender and level of education. The most important determinants of PSD for each group were included in a final model (selected determinants). Eventually selected determinants of PSD are printed in bold.

					Regression analyses performed for:								Final regression analysis			
	Psychologic-personal factors I				Psychologic-personal factors II				Environmental factors							
Determinants of PSD	Importance	Rank	R2 Adjusted	R2	Importance	Rank	R2 Adjusted	R2	Importance	Rank	R2 Adjusted	R2	Importance	Rank	R2 Adjusted	R2
Age	0.007	10	0.473	0.488	0.002	12	0.463	0.492	0.003	15	0.387	0.422				
Level of education	0.001	12	0.473	0.490	0	14	0.461	0.492	0.003	13	0.387	0.419				
Gender	0.010	8	0.46	0.474	0.010	8	0.466	0.484	0.008	10	0.347	0.373				
Disease duration	0.012	7	0.455	0.468									0.004	22	0.615	0.651
No visits to a medical doctor in the past 12 months	0.041	5	0.423	0.435									0.010	15	0.593	0.621
**Comorbidities (SCQ Index)**	**0.113**	**2**	**0.336**	**0.340**									**0.054**	**3**	**0.428**	**0.434**
Quality of life (WHOQOL)	0.049	4	0.414	0.426									0.012	12	0.583	0.607
**In general, how would you rate your health today?**	0.160	**1**	**0.25**	**0.254**									**0.073**	**2**	**0.377**	**0.383**
General living situation	0.008	9	0.467	0.481												
Current working situation	0.032	6	0.443	0.456									0.007	17	0.606	0634
Caregiver role	0.003	11	0.474	0.489												
**Stressful life events [27]**	**0.061**	**3**	**0.389**	**0.400**									**0.015**	**10**	**0.574**	**0.596**
Personality (BFI-10)					0	13	0.463	0.492								
Resilience (Resilience scale)					0.011	7	0.461	0.479					0.009	16	0.601	0.629
**Self-worth**					**0.069**	**2**	**0.357**	**0.363**					**0.036**	**4**	**0.459**	**0.468**
Sense of belonging					0.005	10	0.464	0.489								
Personal beliefs					0.01	9	0.465	0.487								
Self-efficacy (find means and ways to get what you want)					0.002	11	0.463	0.491								
Self-efficacy (deal efficiently with unexpected events)					0.014	6	0.458	0.476					0.006	18	0.605	0.636
**Self-esteem**					**0.046**	**4**	**0.423**	**0.435**					**0.019**	**7**	**0.531**	**0.546**
**Adaptation**					**0.186**	**1**	**0.284**	**0.287**					**0.114**	**1**	**0.286**	**0.289**
**Acceptance of close persons**					**0.048**	**3**	**0.393**	**0.403**					**0.022**	**6**	**0.508**	**0.521**
**Change in personality**					**0.045**	**5**	**0.449**	**0.463**					**0.019**	**8**	**0.541**	**0.558**
Side effects of medication									0.038	3	0.236	0.247	0.010	14	0.593	0.620
Medication (+)^§^									0.007	11	0.37	0.398	0.000	24	0.618	0.656
Medication (-)^#^									0.001	20	0.401	0.448				
Built environment (+)									0.001	18	0.397	0.439				
**Built environment—influence (-)**									**0.058**	**2**	**0.187**	**0.193**	**0.013**	**11**	**0.581**	**0.605**
Public transportation (+)									0	24	0.401	0.458				
Public transportation (-)									0.004	12	0.37	0.401				
Weather or climate (+)									0.009	9	0.34	0.366	0.005	20	0.607	0.641
**Weather or climate (-)**									**0.028**	**6**	**0.323**	**0.341**	**0.017**	**9**	**0.559**	**0.577**
Awareness of health condition people around one has (+)									0.002	17	0.399	0.438				
Awareness of the health condition people around one has (-)									0.033	5	0.307	0.322	0.011	13	0.588	0.614
Help and assistance from family (+)									0.001	21	0.403	0.452				
Help and assistance from family (-)									0.025	7	0.33	0.351	0.003	23	0.614	0.651
Help and assistance from friends (+)									0	23	0.402	0.456				
Help and assistance from friends (-)									0.035	4	0.283	0.296	0.005	21	0.613	0.648
Help and assistance of peers or colleagues (+)									0.002	16	0.393	0.43				
Help and assistance of peers or colleagues (-)									0.003	14	0.387	0.421				
Care from health professionals (+)									0.001	22	0.403	0.455				
Care from health professionals (-)									0.025	8	0.339	0.362	0.006	19	0.605	0.637
Health problems of family members (+)									0.001	19	0.400	0.444				
**Health problems of family members (-)**									**0.065**	**1**	**0.106**	**0.11**	**0.025**	**5**	**0.489**	**0.499**
Correlation between predicted and obtained values	***0*.*76***				***0*.*74***				***0*.*68***				***0*.*85***			

^§^ (+): positive influence of determinant on PSD.

^#^ (-): negative influence of determinant on PSD.

## Discussion

In the present work, we determined a brief set of environmental and psychologic-personal factors that are determinants of PSDs experienced by persons with brain disorders. To our knowledge, this is the first work specifically targeting determinants of PSD looking for determinants valid across brain disorders. The identified determinants, namely comorbidities, personal appraisal of health status, stressful life events, personality, adaptation, self-esteem, self-worth, built environment, weather and health problems of family members provide useful information to introduce the implementation of cross-cutting interventions, social actions and policy tools to lower PSD experienced in daily life by persons with brain disorders. This set can also be used to complement a recently proposed reliable and valid direct metric of PSD for brain disorders called PARADISE24 [13].

The advantage of the present work is its use of a comprehensive definition of both PSD experienced by persons with brain disorders and its determinants. Relying on the biopsychosocial framework proposed by the WHO, we understand PSD (also referred to as “burden”), as impairments in mental functions and body functions under nervous-system control, activity limitations and participation restrictions resulting from the interaction between the brain disorder, environmental and personal factors. The diversity of the determinants identified as the most relevant in our work, namely the negative impact of the weather, the built environment and the presence of health problems among family members, might sound peculiar at first glance. They coincide with the broad definition of environmental factors proposed in the ICF, which includes products and technology (like medication or built environment), the natural environment, compromising weather conditions, social and professional support and attitudes and services, systems and policies. This broad conceptualization implies a large range of intervention targets to impact PSD, moving the focus from exclusively curative or symptom-oriented strategies to strategies that target changing or adapting the environment, which is a common strategy in degenerative diseases, but still a rare approach in brain disorders.

The added value of identifying the negative impact of weather or climate, which encompasses temperature, humidity and precipitation, among other factors, and the negative impact of the built environment, which includes design, construction and building of entrances, exits, and facilities, as strong predictors of the experienced burden directs the attention of policy makers to the need for environmental interventions and adaptations for individuals with brain disorders. The need to adapt the built environment is very intuitive in disorders leading to wheelchair use, as is the case in MS or PD [28]. Our results point out, however, that the built environment also exerts a significant negative impact on other brain disorders, such as dementia, depression and substance dependence [9]. Our hypothesis is that the interplay between PSD experienced by persons with brain disorders (e.g., cognitive problems, low energy levels and pain) and the built environment is as important as its interplay with limitations in mobility. The impact of weather or climate on PSD has been examined and corroborated in relation to behavioural and psychological problems in dementia [29], and psychotic exacerbation in schizophrenia [30]. It is also thought to trigger migraines [31] and influence admission rates for affective disorders [32] and disease progression in MS [33]. In our previous study, rates of patients considering the weather or climate to have an impact on PSD ranged from 61% in substance dependence, 69% in dementia, 78% in schizophrenia and up to 90% in migraine [9]. The present study stresses the negative impact of these determinants on PSD, since the questions targeting a potential positive impact of weather and built environment did not remain in the final regression model. The specific features of the built environmental and weather negatively impacting PSD cannot, however, be fully explained by our data, and corresponding qualitative studies among persons with brain disorders are lacking. A better understanding of the needs of persons with brain disorders regarding built environmental and weather is required for adequate policies to be developed and implemented.

The third determinant identified refers to the presence of health problems of family members. In our study, this environmental factor was pointed out as a determinant by 50% or more persons with migraine, dementia, depression, schizophrenia and substance dependence. A hypothesis that might explain this result is that social support on the part of the family, i.e. emotional and financial support, advocacy and housing, which has been recognized as an important protective factor in depression [34] and schizophrenia [35] is threatened if family members are themselves ill. In the light of our results, the assessment of the presence of health problems among family members is highly recommended to clinicians and health professionals involved in the treatment of persons with brain disorders and interested in reducing their experienced burden. It should be taken in account in clinical work and the community should provide enough support for these families.

The conceptualization of determinants used in our work goes beyond environmental factors and includes personal and psychological factors as important determinants of PSD. The four psychological variables identified as determinants encompass changes in personality, adaptation on the part of affected persons and persons close to them, self-esteem and self-worth. The key role played by psychological features in the experienced burden of persons with brain disorders, for example in self-esteem, has been extensively reported in the literature [36, 37]. The added value of our work here is to show that these psychological features are relevant across brain disorders. Especially adaptation, i.e. whether the person has learned to live with the problems and difficulties associated with his or her brain disorder and whether the person thinks that the people around him/her have come to terms with their problems and difficulties, were very important determinants of the experienced burden across disorders. Our findings have significant implications for clinicians and health professionals and stress the importance of providing psychological and psychosocial treatment and encouraging participation in self-help groups and patients’ organizations to emphasise adaptation in the absence of curative strategies and improve self-esteem and self-worth among persons with brain disorders in general. Our work stresses the importance of existing disease-specific strategies and encourages the development of cross-cutting strategies for brain disorders in general.

The three psychologic-personal factors identified in our work as determinants of PSD across brain disorders are not surprising from a disease-specific perspective, but highly important from the perspective of so-called “horizontal epidemiology”. It is well known that comorbidities are highly prevalent in persons with brain disorders, which has been extensively discussed in the WHO’s initiative “No Health without Mental Health”. This is especially important because the provision and the quality of care that people with mental disorders receive is worse than in the general population [1]. Stressful life situations, an established risk factor and trigger for the development of several conditions, especially mood disorders [38], as well as the subjective appraisal of one’s own health status, which is an acknowledged powerful predictor of mortality [39], were also identified as valuable predictors of PSD in brain disorders. We therefore emphasise the importance of a careful assessment of comorbidities and stressful life situations and the need for sound disease-management strategies in all brain disorders. Moreover, our work discloses that the subjective appraisal of health status, a frequently-used predictor of mortality, can also be used by researchers to predict the experienced burden in brain disorders.

Our study has three strengths worth mentioning. First, we relied on the recently challenged and confirmed hypothesis of horizontal epidemiology, namely that PSDs experienced in daily life by persons with brain disorders are similar across conditions because they are understood as the outcome of the interaction between the disorder and environmental and personal factors. The fact that PSDs—in terms of burden—are shared supported the idea of looking for a set of common determinants of PSD across brain disorders. Preliminary research going beyond a disease-specific approach and looking at determinants across disorders supports our findings, so that we definitely recommend further studies using the same perspective. A direct and hugely important consequence of more knowledge about common determinants of PSD is the development of cross-cutting interventions and universal policy strategies. Second, we are targeting not determinants of the development of disorders, which has already been extensively carried out, but determinants of PSD experienced in daily life by persons living with brain disorders. Knowledge of determinants, risk factors and the aetiology of brain disorders has been continuously expanded, for instance by carrying out life-course, risk-modelling exercises [40] or focusing on the interactions among determinants during life stages [41, 42]. Although such efforts are undeniably valuable to develop primary prevention strategies, the high prevalence of brain disorders has a huge impact on daily life [1] and urgently requires research on determinants which can be targeted to alleviate the experienced burden of the population currently affected by brain disorders. Third, this set of 11 determinants is suitable to complement a recently proposed reliable and valid direct metric of PSD in brain disorders called PARADISE24. To become a comprehensive tool corresponding with horizontal epidemiology, authors stress the need to add key determinants to this metric, like environmental and psychologic-personal factors, which interact with brain-disorder and are determinants of PSDs. Our set provides these key determinants.

Our findings have to be viewed in the light of their limitations. First, we used a convenience sample of persons with brain disorders, so the generalizability of our results is unclear. Studies with larger and representative samples, especially taking into account the inclusion of persons with different disease severity, are required to confirm our results. Second, we collected information during an interview, and this might have influenced the answers to sensitive topics, such as the positive and negative influence of the access to illegal drugs or alcohol on the experienced PSD. We might have missed important determinants sensitive to social-desirability bias. Finally, we collected information on the direction (positive or negative) and the extent of this influence using the response options none, mild, moderate or strong on the determinants on the PSD. It is not possible to further disclose how determinants impact PSDs or which determinants impact which specific PSD. In the scope of PARADISE we have published a qualitative study exploring the experiences of people with seven brain disorders regarding PSDs and determinants of PSD, which can be seen as complementary to the present work [11]. Since the knowledge coming from qualitative studies is essential to develop interventions, we strongly encourage researchers to embark in future similar qualitative studies including further brain disorders.

In summary, we identified a brief set of environmental and personal determinants of PSD experienced by persons with brain disorders. We recommend that this set be used together with the PARADISE24 metric to provide a comprehensive picture of the experienced burden and its determinants. The routine inclusion of our set in data collection is a first step towards expanding the knowledge about determinants of PSD across brain disorders and might be used to guide health professionals, policy makers, affected persons and their families in the development and implementation of effective interventions or actions to reduce the burden experienced by persons with brain disorders. Since our data does not provide a specific understanding of how determinants like built environment and weather influence PSD, we encourage researchers to carry out qualitative studies to further explore these interactions.
