# Correction: Determinants of Psychosocial Difficulties Experienced by Persons with Brain Disorders: Towards a 'Horizontal Epidemiology' Approach

**DOI:** 10.1371/journal.pone.0146713

**Published:** 2016-01-05

**Authors:** The PLOS ONE Staff

The affiliation for the fourteenth author is incorrect. Anna Chrostek is not affiliated with #5 but with #6, Department of Psychiatry, Institute of Psychiatry and Neurology, Warsaw, Poland.

In addition, the eleventh author’s name is misspelled in the PDF version of the article. The correct name is: Piotr Świtaj. The publisher apologizes for the error.
